# Early Initiation of Enzyme Replacement Therapy in Infantile Onset Pompe Disease Improves Cardiac Outcomes: A Longitudinal Analysis

**DOI:** 10.1002/jmd2.70060

**Published:** 2026-01-23

**Authors:** Jennifer L. Cohen, M. Makenzie Beaman, Eleanor Rodriguez‐Rassi, V. Grace Stafford, P. Brian Smith, Andrew P. Landstrom, Priya S. Kishnani

**Affiliations:** ^1^ Department of Pediatrics, Division of Medical Genetics Duke University Durham North Carolina USA; ^2^ Department of Pediatrics, Division of Neonatology Duke University Durham North Carolina USA; ^3^ Department of Pediatrics, Division of Cardiology Duke University Durham North Carolina USA

**Keywords:** arrhythmia, cardiomyopathy, echocardiogram, electrocardiogram, infantile onset Pompe disease

## Abstract

The objective of this study is to evaluate whether early enzyme replacement therapy (ERT) initiation is associated with a lower incidence of echocardiogram abnormalities and cardiac conduction abnormalities compared to later ERT initiation. We identified a cohort of patients treated with ERT for infantile onset Pompe disease (IOPD) and evaluated their cardiac outcomes by comparing clinically collected longitudinal functional echocardiogram and electrocardiogram (EKG) data. Longitudinal mixed‐effects analysis was used to compare cardiac outcomes among the cohort based on timing of ERT initiation as a continuous variable (months of age) and accounted for repeated measures in an individual patient. Time‐to‐event analysis and Cox regression were performed to evaluate the time to achievement of normal left ventricular mass index (LVMI) based on ERT initiation as a dichotomous variable (≤ 1 month versus > 1 month of age). Early treatment was associated with significant improvements in cardiac remodeling as demonstrated by multiple cardiac parameters with better outcomes based on earlier treatment such as interventricular septum thickness in diastole and systole, left ventricular posterior wall thickness in diastole and systole, and biventricular hypertrophy. The early‐treated cohort (those started on ERT ≤ 1 month of age) achieved a normal LVMI faster compared to late‐treated patients. Early treatment with ERT in patients with IOPD leads to improved cardiac chamber dimension parameters. Treatment initiation ≤ 1 month of age can shorten the time to achieve a normal LVMI. Our findings were limited by the nature of the data collection, which was retrospective and clinically driven; the results presented in this study, however, support the clinical importance of early therapeutic intervention in IOPD. Early initiation with ERT in patients with IOPD can shorten the time to achieve a normal LVMI and can improve cardiac chamber dimension parameters.

## Summary

- Early initiation with enzyme replacement therapy in patients with IOPD can shorten the time to achieve a normal LVMI and can improve cardiac chamber dimension parameters.
- Early treatment was associated with significant improvements in cardiac remodeling as demonstrated by multiple cardiac parameters with better outcomes based on earlier treatment such as interventricular septum thickness in diastole and systole, left ventricular posterior wall thickness in diastole and systole, and biventricular hypertrophy.

## 1 Introduction

Pompe disease is characterized by a pathologic accumulation of lysosomal glycogen due to acid alpha‐glucosidase (GAA) enzyme deficiency. Genotype determines the expected residual enzyme activity and severity of disease, including disease classification (infantile vs. late onset) and cross‐reactive immunologic material (CRIM) status. CRIM‐negative status portends the most severe disease phenotype.

In patients with infantile onset Pompe disease (IOPD), the cardiac muscle is particularly affected, and individuals often have evidence of cardiomyopathy at birth or even prenatally. The categorization of infantile onset Pompe disease (compared to late‐onset Pompe disease) includes the presence of cardiomyopathy in the first year of life. Without newborn screening (NBS), individuals are often diagnosed later in infancy, following symptomatic presentation [1]. In IOPD, progressive hypertrophic cardiomyopathy and respiratory insufficiency lead to early mortality, typically by age 2 years if untreated [1]. Known cardiac manifestations include electrocardiographic (EKG) findings of a shortened PR interval, large amplitude R wave, delta waves suggestive of Wolff‐Parkinson‐White (WPW) ventricular preexcitation pattern (a type of supraventricular tachycardia), wide QRS duration, and increased QT dispersion (QTd), large left ventricular (LV) voltages (SV1 + RV6), and cardiomegaly [2, 3, 4]. Recent studies have additionally noted long or borderline QT intervals, first‐degree heart block, and atrioventricular nodal reentrant tachycardia [5]. Hypertrophic cardiomyopathy and ventricular arrhythmias [6, 7, 8, 9] represent a significant disease burden. Enzyme replacement therapy (ERT) efficacy studies from the early clinical trials showed that treatment leads to an increase in PR interval, a decrease in QTd and LV voltage, and no change to QTc interval [2].

ERT typically reverses cardiac hypertrophy, yet the prolonged duration of time to normalization of left ventricular mass index (LVMI) remains a concern. Furthermore, in the setting of high sustained anti‐drug antibody titers (HSAT), a seemingly resolved cardiac phenotype can reappear [5]. ERT does not efficiently clear glycogen from the conduction tissue cells, which become enlarged in Pompe disease [10] and may lead to a possible antegrade accessory pathway that could serve as a substrate for supraventricular tachycardia [7]. Furthermore, the ventricular septum hypertrophy causes abnormalities in the atrioventricular conducting system [10]. A recent study utilized myocardial deformation analysis in conjunction with conventional echocardiography to follow patients treated with ERT to better understand cardiac function; the investigators demonstrated restoration of normal cardiac function after 1 year of ERT and stability thereafter [11]. The median age at start of ERT was 3.2 months with ERT initiation timing not investigated as a factor [11].

Pompe disease was added to the Recommended Uniform Screening Panel (RUSP) for NBS in the United States in 2015 with a purpose of identifying and treating patients prior to extensive symptom onset [12]. Prior studies have demonstrated improved overall clinical outcomes with screening and earlier treatment [13, 14, 15, 16], however, these studies did not investigate detailed echocardiogram measures or detailed EKG measures. There is a critical need to comprehensively study these parameters among a genetically diverse population.

In the present study, we sought to determine the impact of ERT initiation timing on longitudinal cardiac outcomes, including exploratory analyses of multiple EKG and echocardiogram parameters. Although the cardiac system readily responds to ERT, delayed treatment initiation can lead to suboptimal outcomes and incomplete resolution of abnormalities. Our study provides further insights into cardiac responses following early treatment.

## 2 Materials and Methods

### 2.1 Study Population

The Duke Pompe database includes 132 patients with IOPD (defined as having cardiac hypertrophy within the first year of life and/or with a genotype known to correspond to an infantile onset phenotype) recruited from within the United States and internationally. Individuals who choose to participate are consented through Duke University's IRB‐approved study, Determination of Cross‐Reactive Immunological Material (CRIM) status and Longitudinal Follow‐up of Individuals with Pompe Disease (Pro00001562). Included individuals have been diagnosed with IOPD based on their genotype and a confirmed phenotype consistent with the diagnostic classification. Individuals are introduced to the Duke natural history study either through receiving clinical care at Duke, receiving care from a local physician seeking expert clinical advice, a clinicaltrials.gov search, or peer referral from within the Pompe patient/family community. International patients are consented in their native language using an interpreter. Data is obtained by the Duke research team by requesting records from treating physicians or requesting that the local physician document key information in an excel file with open fields. A de‐identified data form exists, as needed, based on a country's medical privacy laws.

For our longitudinal and retrospective study, individuals were selected for inclusion if there were sufficient data for that individual (Table 1). If the patient did not have critical data such as ERT initiation date, immune tolerance induction (ITI) administration documentation, or CRIM status, they were excluded from the study. We therefore conducted a comprehensive assessment of 55 patients' longitudinal functional echocardiogram and EKG measures. Patients were diagnosed through prenatal testing, newborn screening, or clinical presentation. Each patient's ERT dosing regimen, ITI administration [17, 18, 19, 20], and presence or absence of HSAT (defined as ≥ 12 800) were also recorded in our data collection (Table 1) but did not factor into the statistical analyses. With a cohort of only 55 patients, we did not have sufficient data to explore how ERT regimen and HSAT are associated with cardiac outcomes. We would not be able to investigate the dichotomous age of treatment initiation (≤ 1 month vs. > 1 month of age) with any other factor due to an insufficient sample size. ERT regimen (see Table 1) is also variable without a clear strategy to combine the ERT regimen categories. Individuals are geographically diverse (recruited throughout the United States and abroad), include both CRIM‐positive and CRIM‐negative status, and have a heterogeneous spectrum of genotypes (Table S1). All data points were collected based on clinically recorded echocardiogram and EKG assessments. Many data points were collected from the same individual and an individual's clinical assessments varied in interval frequency and the total number of assessments.

**TABLE 1:** Patient characteristics by treatment timing group.

Characteristic	Late (*n* = 45)	Early (*n* = 10)
ERT regimen, *n* (%)		
20 mg/kg every other week	17 (37.8)	4 (40.0)
40 mg/kg weekly	13 (28.9)	1 (10.0)
40 mg/kg every other week	3 (6.7)	1 (10.0)
20 mg/kg weekly	3 (6.7)	1 (10.0)
43 mg/kg weekly	0 (0.0)	1 (10.0)
19.5 mg/kg every other week	1 (2.2)	0 (0.0)
5 mg/kg twice weekly	1 (2.2)	0 (0.0)
Not available/recorded	7 (15.6)	2 (20.0)
Immunomodulation, *n* (%)		
Received ITI^a^	31 (68.9)	9 (90.0)
No ITI	14 (31.1)	1 (10.0)
CRIM status, *n* (%)		
CRIM‐positive	25 (55.6)	8 (80.0)
CRIM‐negative	20 (44.4)	2 (20.0)
High antibody titers, *n* (%)		
No high titers	32 (71.1)	7 (70.0)
High titers present	9 (20)	1 (10.0)
Unknown	4 (8.9)	2 (20.0)
Baseline LVMI (g/m^2^), median (range)	221 (64–628.6)	112 (55.5–157)
Age at ERT initiation (months), median (range)	4.2 (1–13.5)	0.5 (0–0.8)

*Note:* Early treatment defined as ERT initiation ≤ 1 month of age; late treatment defined as ERT initiation > 1 month of age. Data are presented as *n* (%) for categorical variables and median (range) for continuous variables. ERT regimen is the initial dosing regimen for each patient.

Abbreviations: CRIM, cross‐reactive immunologic material; ERT, enzyme replacement therapy; ITI, immune tolerance induction.

^a^ 5 of these patients were treated with rescue ITI (either in addition to or instead of prophylactic ITI) and 4/5 were late treated; 3/5 were CRIM positive.

For some analyses, patients were stratified into two groups based on age at ERT initiation: early treatment (≤ 1 month of age, *n* = 10) and late treatment (> 1 month of age, *n* = 45). For other analyses, age at ERT initiation (months) was used as a continuous variable. Age was calculated as the difference between date of birth and date of ERT initiation in days, divided by 30.45 (average number of days in a month). Patient demographic and clinical characteristics were summarized by the dichotomous treatment timing group (early vs. late).

A total of 360 clinical assessments were analyzed, with 55 from early‐treated patients and 305 from late‐treated patients. Patients were also stratified by CRIM status: positive (*n* = 33) or negative (*n* = 22). Among the early‐treated group, 8 were CRIM‐positive and 2 were CRIM‐negative, while in the late‐treated group, 25 were CRIM‐positive and 20 were CRIM‐negative. Of the 360 assessments, 208 were from CRIM‐positive patients and 152 were from CRIM‐negative patients.

The mean age of ERT initiation with alglucosidase alfa was 0.45 months (range 0.03–0.76) in the early‐treated cohort and 5.05 months (range 1.02–13.5) in the late‐treated cohort. The mean age at first assessment (electrocardiogram or echocardiogram) was 0.27 months (range 0–0.69) for the early‐treated group and 5.99 months (range 0.1–27.49) for the late‐treated group. The mean age at last assessment was 21.2 months (range 3.58–47.32) for the early‐treated group and 40.88 (range 4.11–202.33) for the late‐treated group. The mean follow‐up duration was 20.92 months (3.09–47.32) for the early‐treated cohort and 34.89 months (0–199.51) for the late‐treated cohort.

### 2.2 Electrocardiogram Data Collection

The data collected and analyzed from the electrocardiograms included: atrial enlargement (Left atrial enlargement if P wave width was > 2.5; right atrial enlargement if P wave height was > 3), PR interval, QRS duration, QT, QTc, right axis deviation, T axis, T wave inversion in inferior, lateral and anteroseptal leads, presence of isoelectric T waves in inferior, lateral and anteroseptal leads, ST elevation in inferior, lateral and anteroseptal leads, ST depression in inferior, lateral and anteroseptal leads. PR interval and QRS interval were recorded from the automated read; QTc interval was manually measured using 3 peaks normalized to RR interval with electronic calipers. Additional parameters investigated were abnormal voltages (greater than normal for age): R wave amplitude in leads V1 and V6, and S wave amplitude in leads V1 and V6. The electrocardiogram traces were also reviewed for signs of hypertrophy and recorded as right ventricular hypertrophy, left ventricular hypertrophy, and biventricular hypertrophy. Age‐dependent intervals, such as PR intervals, were recorded as either normal, shortened, or elongated based on the patient's age at the time of the EKG and age‐related norms. Data (text) regarding automated read EKG abnormalities were collected by manual review of EKGs. EKGs were reviewed by MMB under the guidance of a pediatric cardiologist (APL) and any uncertain findings were specifically reviewed by the pediatric cardiologist (APL).

### 2.3 Echocardiogram Data Collection

The data collected from the echocardiograms included LVMI, ejection fraction, shortening fraction, and Z‐scores for the following: interventricular septum thickness in diastole and in systole (IVSd and IVSs); left ventricular internal diameter in diastole and in systole (LVIDd and LVIDs); and left ventricular posterior wall thickness in diastole and in systole (LVPWd and LVPWs). Additionally, the presence of tricuspid valve insufficiency, pulmonary valve stenosis, and pulmonary valve insufficiency were recorded and analyzed. If there was no LVMI in the echocardiogram report, the LVMI was calculated by dividing the LVM by the patient's body surface area (BSA) at the time of the echocardiogram. LVMI values were also converted to g/m^2^ if they were recorded in g/ht^2.7^. LVMI was defined as increased if it was ≥ 64 g/m^2^; two standard deviations above the norm (48.8 ± 8) [21]. Ejection fraction was recorded as normal (> 55%) or abnormal (< 55%). Shortening fraction was also recorded as normal (> 30%) or abnormal (< 30%) in a binary fashion.

### 2.4 Statistical Analyses

We stratified the children by ERT initiation timing and compared those who began ERT early (defined as ≤ 1 month of age) to those who began treatment later. We sought to test the hypothesis that early postnatal initiation of ERT would normalize cardiac hypertrophy as measured by LVMI more rapidly. Upon review, patients in the early‐treated cohort were diagnosed early following either (a) a positive family history in a sibling (and thus diagnosed prenatally) or (b) following a positive NBS result; this unbiased approach ensures that those treated at ≤ 1 month of age were not treated earlier because of a more severe symptomatic presentation. All clinically diagnosed patients presented after 1 month of age and were therefore treated at > 1 month of age (which we defined as late treatment). We also investigated ERT initiation timing as a continuous variable to conduct exploratory analyses regarding functional cardiac endpoints and electrocardiogram abnormalities.

### 2.5 Time‐To‐Event Analysis

For the hypothesis‐driven question regarding LVMI, we conducted a time‐to‐event analysis to evaluate the time to LVMI normalization using Kaplan–Meier curves stratified by treatment timing group and included LVMI baseline and CRIM status as covariates. Baseline refers to a subject's first recorded LVMI. This analysis included 39 patients: 16 CRIM negative (1 early‐treated; 15 late‐treated) and 23 CRIM positive (7 early‐treated; 16 late‐treated) across 17 events (achievement of normal LVMI). Two patients were excluded from this analysis since they had a normal LVMI at baseline (2 CRIM negative patients: 1 early‐treated and 1 late‐treated). For the late‐treated patient, the baseline LVMI that is available for our dataset was recorded after 10 months of treatment with ERT; although the LVMI was normal at this timepoint, free text recorded “Severe LV hypertrophy.” For the early‐treated patient, the time of the normal baseline LVMI was at 1 day of life and treatment was started at day 4 of life. Both patients have IOPD based on a confirmed genotype consistent with IOPD. Cox regression analysis was performed to determine the hazard ratio and confidence interval. We performed the same model but with age at ERT initiation as a continuous variable rather than a dichotomous variable (model not shown).

We then separated CRIM positive patients from CRIM negative patients to run these same time‐to‐event and Cox regression analyses, but since there was only 1 CRIM negative patient in the early‐treated cohort who also had an abnormal baseline LVMI, and only 2 CRIM negative patients in the early‐treated cohort in general, only the CRIM positive patient cohort was able to be analyzed separately. Kaplan–Meier curves for time to LVMI normalization and a Cox regression model, controlling for baseline LVMI, was conducted. This analysis included 23 patients (7 early‐treated; 16 late‐treated) and 9 LVMI normalizations achieved.

### 2.6 Continuous Cardiac Outcomes and Binary Cardiac Outcomes Analysis

To investigate the association between age at ERT initiation and various cardiac outcomes, we used mixed‐effects regression models that appropriately accounted for repeated measurements within individuals by using random intercepts for patient identification number. When mixed‐effects models resulted in singular fits due to insufficient between‐patient variance, simple linear regression (for continuous outcomes) or logistic regression (for binary outcomes) was employed as a fallback approach. All models were adjusted for CRIM status and time post‐ERT initiation.

For continuous cardiac parameters (e.g., LVMI, chamber dimension Z‐scores), linear mixed‐effects models were fit using the lme4 package in R, with the cardiac measure as the dependent variable. Age at ERT initiation (in months) was modeled as a continuous fixed‐effect predictor. Models were adjusted for clinical covariates including CRIM status (positive vs. negative) and time elapsed post‐ERT at the time of cardiac assessment. Patient identifier was included as a random intercept to account for within‐subject correlation due to repeated observations.

For binary cardiac outcomes indicating the presence or absence of specific pathological features (e.g., ventricular hypertrophy, conduction abnormalities), mixed‐effects logistic regression models were similarly constructed with the same fixed and random effect structure. Model fitting was performed using maximized likelihood methods with appropriate optimizers to ensure convergence.

Effect estimates for continuous outcomes were reported as the change in the cardiac parameter per 1 month increase in age at ERT initiation. For binary outcomes, age at ERT initiation was included as a continuous predictor in mixed‐effects logistic regression models; the results are presented as odds ratios (ORs) with 95% confidence intervals, reflecting the change in the odds of the outcome associated with each additional month of delay in starting ERT.

Given the known prognostic importance of CRIM status in IOPD, we used two complementary approaches to determine whether CRIM status influences cardiac outcomes and modifies treatment timing effects. First, we tested for main effects of CRIM status by including it as a covariate in our mixed‐effects models. Second, we conducted stratified analyses by CRIM status, fitting separate models within CRIM positive and CRIM negative patient groups to compare treatment timing effects between these CRIM subtypes. This stratified approach was used after convergence issues were observed with an attempted interaction testing; our approach provides direct estimates of treatment timing effects within each CRIM subgroup.

### 2.7 EKG Automated Abnormalities

All available EKGs were reviewed, and automated findings were recorded and reviewed. Since the majority of abnormalities were not considered to be clinically significant (arrhythmias), we did not conduct further analyses on these data.

For all analyses, statistical significance was defined as *p* < 0.05. All analyses were performed using R studio version 4.4.1 (2009–2024 Posit Software, PBC). The lme4 package was used for mixed models and ggplot2 was used for visualization. Artificial Intelligence Generated Content (AIGC) tools, specifically Claude by Anthropic, and DukeGPT were used to generate R code and assisted in drafting the statistical analysis methodology and interpretation of analyses (relevant to the methods and results sections of this manuscript).

## 3 Results

### 3.1 Patient Characteristics

Table 1 details the results of the cohort's characteristics as it relates to CRIM status, receipt of immunomodulation, ERT dosing regimen, development of HSAT, baseline LVMI, and median age of ERT initiation in early and late treated cohorts. Of note, 22 patients experienced a change in ERT dosing regimen. Thirty‐seven individuals in the total cohort received prophylactic ITI; 5 individuals received rescue ITI (either in addition to or instead of prophylactic ITI) within the time of data analysis. Of these 5, 4/5 were in the late treatment cohort and 3/5 were CRIM positive.

### 3.2 Time‐To‐Event Analysis

**FIGURE 1:**
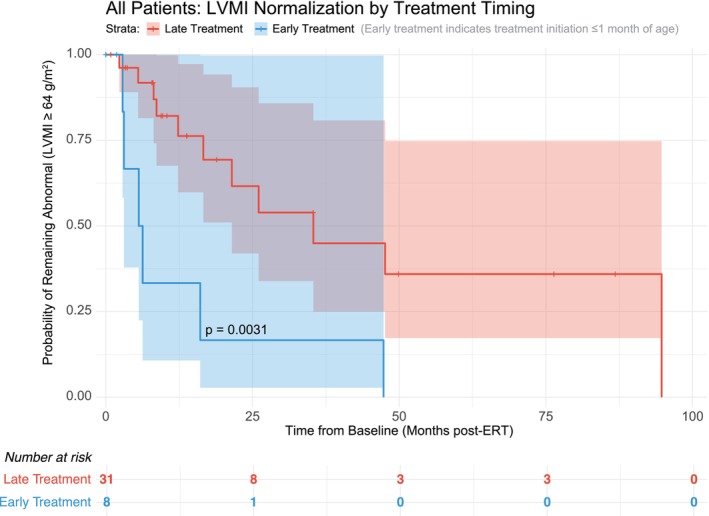
Kaplan–Meier survival curve showing the probability of remaining with an abnormal LVMI (≥ 64 g/m^2^) over time since baseline LVMI recording (with time reported as months post‐ERT) among patients with abnormal baseline LVMI, stratified by treatment timing (early vs. late). Patients are grouped based on whether they received early or late enzyme replacement therapy (ERT) initiation. The analysis accounts for the time to LVMI normalization as the event of interest. Shaded areas represent 95% confidence intervals, and a risk table displays the number of patients at risk at each time interval. The log‐rank test demonstrated a statistically significant difference in LVMI normalization between early and late treatment groups (*p* = 0.0031), indicating that earlier initiation of ERT is associated with faster normalization of LVMI.

Among 39 individuals included in the time‐to‐event analysis, mean LVMI baseline was 208.5 g/m^2^, mean age at ERT initiation was 3.8 months, and mean follow‐up time was 33 months. Log rank test comparing survival curves between early and late treatment groups showed *p*‐value: 0.0031 (Figure 1). Cox regression analysis was performed to determine the hazard ratio (3.675) and confidence intervals (1, 13.507), *p* = 0.05 comparing early vs. late treatment initiation, adjusted for CRIM status and baseline LVMI. We performed the same model but with age at ERT initiation as a continuous variable and this did not quite reach statistical significance. We therefore chose to report the dichotomous treatment timing model for this analysis.

**FIGURE 2:**
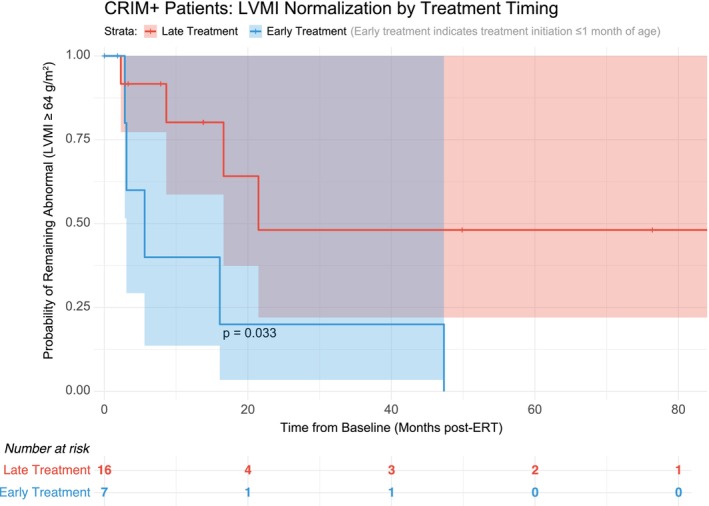
Kaplan–Meier survival curve depicting the probability of remaining with an abnormal LVMI (≥ 64 g/m^2^) over time since baseline LVMI recording (with time reported as months post‐ERT) among CRIM‐positive patients. Patients are grouped by treatment timing into early and late initiation of enzyme replacement therapy (ERT). Shaded areas represent 95% confidence intervals around the survival estimates. The risk table below the plot displays the number of patients at risk at each time point. The log‐rank test comparing early versus late treatment groups revealed a statistically significant difference (*p* = 0.0327), indicating that earlier ERT initiation is associated with faster normalization of LVMI among CRIM‐positive patients.

Among 23 CRIM positive patients in the time‐to‐event analysis, all patients in this sub‐population had abnormal LVMI at baseline and 9 events of LVMI normalization were observed. The mean baseline LVMI was 200.6 g/m^2^. Log‐rank test *p*‐value is 0.0327 (Figure 2). The Cox Regression hazard ratio is 2.276 (CI: 0.507, 10.213), *p* = 0.283.

Three individuals demonstrated LVMI normalization with a recurrence of abnormal elevation. A CRIM negative patient in the late treatment cohort achieved normalization at 9.82 months of age (7.39 months after ERT initiation) and then at 11.99 months demonstrated recurrence of elevated LVMI, which remained the case through the patient's last assessment at 89.72 months. A CRIM positive patient in the late‐treated cohort achieved LVMI normalization at 11.46 months of age (8.41 months after ERT initiation) and then at 202.33 months showed elevation of LVMI. Unfortunately, we do not have records in between these two assessments. A CRIM positive patient in the early treated cohort achieved LVMI normalization at 6.14 months of age (5.39 months after ERT initiation) and then at 9.46 months had abnormal elevated LVMI and remained elevated at 13, 19, 28, and 33 months' assessments with a return to normalization at 45.52 months of age. None of these three patients had high anti‐drug antibody titers. All other subjects in the cohort with normalized LVMI remained normalized through their last recorded assessment.

### 3.3 Continuous and Binary Cardiac Outcomes

**FIGURE 3:**
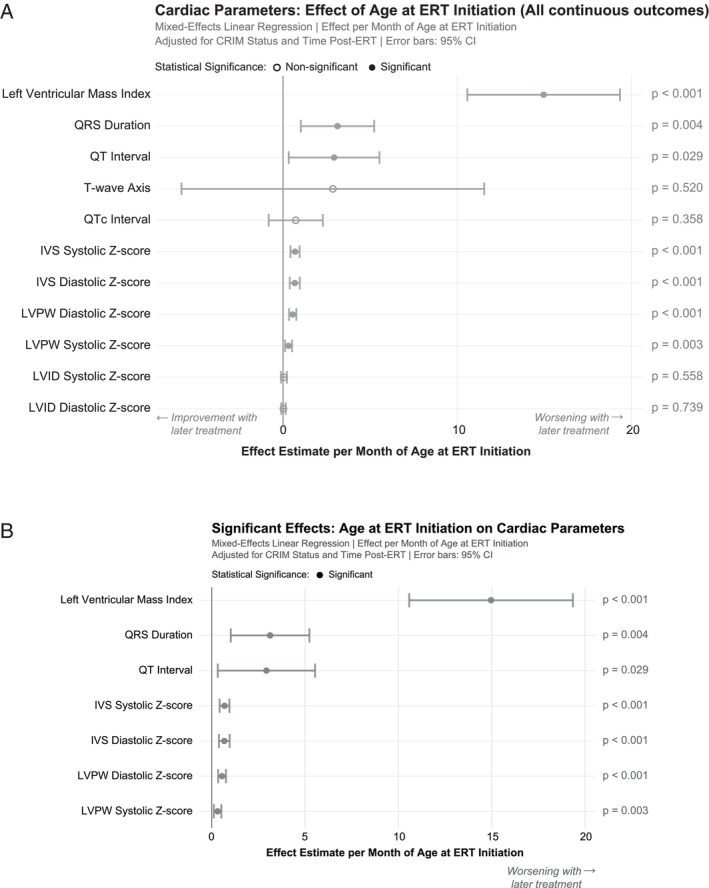
(A) Forest plot showing the effect of age at ERT initiation (as a continuous variable) on all results of continuous cardiac outcomes, adjusted for CRIM status and time post‐ERT initiation at the time of cardiac outcome data collection point. IVS, interventricular septum thickness; LVPW, left ventricular posterior wall thickness; LVID, left ventricular internal diameter. (B) Forest plot showing the effect of age at ERT initiation (as a continuous variable) on statistically significant results of continuous cardiac outcomes, adjusted for CRIM status and time post‐ERT initiation at the time of cardiac outcome data collection point. IVS, interventricular septum thickness; LVPW, left ventricular posterior wall thickness.

**FIGURE 4:**
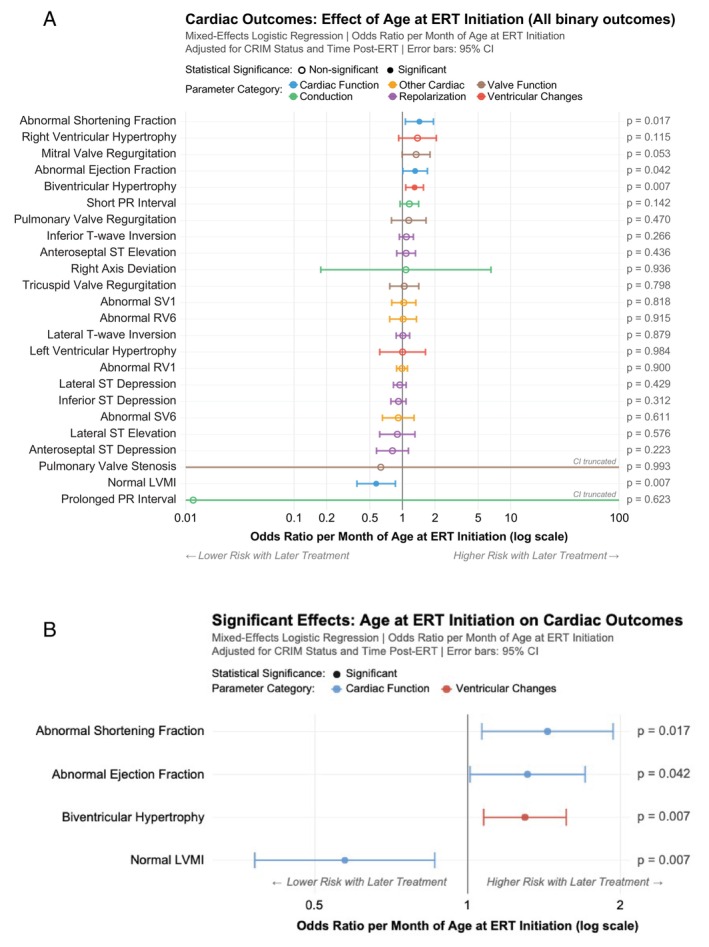
(A) Forest plot showing the effect of age at ERT initiation (as a continuous variable) on all results of binary cardiac outcomes, adjusted for CRIM status and time post‐ERT initiation at the time of cardiac outcome data collection point. Abnormal SV1, abnormal S wave amplitude in lead V1; Abnormal RV6, abnormal R wave amplitude in lead V6; Abnormal RV1, abnormal R wave amplitude in lead V1; Abnormal SV6, abnormal S wave amplitude in lead V6; LVMI, left ventricular mass index. (B) Forest plot showing the effect of age at ERT initiation (as a continuous variable) on statistically significant results of binary cardiac outcomes, adjusted for CRIM status and time post‐ERT initiation at the time of cardiac outcome data collection point. LVMI, left ventricular mass index.

Early ERT initiation was associated with significant improvements across multiple cardiac parameters (Figures 3A,B and 4A,B). Of the 11 continuous cardiac outcomes analyzed, 7 were statistically significant. Of the 24 binary outcomes analyzed, 4 were statistically significant. Statistically significant effects included the following: each month of treatment delay increases LVMI by 14.9617 units, on average (*p* < 0.001); each month delay increases IVSd Z‐score by 0.6738 units, on average (*p* < 0.001); each month delay increases IVSs Z‐score by 0.687 units, on average (*p* < 0.001); each month delay increases LVPWd Z‐score by 0.5534 units, on average (*p* < 0.001); and each month delay increases LVPWs Z‐score by 0.3145 units, on average (*p* = 0.003); each month delay increases QRS duration by 3.1257 units, on average (*p* = 0.004); each month delay increases QT interval by 2.9311 units, on average (*p* = 0.029). Statistically significant binary effects included the following: each month delay increases odds of biventricular hypertrophy by 29.6% (OR = 1.296, *p* = 0.007); each month delay increases odds of an abnormal shortening fraction by 43.6% (OR = 1.436, *p* = 0.017); each month delay increases odds of an abnormal ejection fraction by 31.2% (OR = 1.312, *p* = 0.042); each month delay decreases odds of a normal LVMI by 42.7% (OR = 0.573, *p* = 0.007).

CRIM status was not statistically associated with cardiac outcomes in the main effects model. In the stratified analysis for CRIM status, three parameters showed a larger treatment timing effect in the CRIM negative cohort: LVMI, IVSd Z‐score, and IVSs Z‐score.

### 3.4 Automated EKG Abnormalities

Upon review with a pediatric cardiologist (APL) of all automated conduction abnormalities reported on the EKGs across the cohort, only one arrhythmia was noted—an idioventricular rhythm, which is a slow form of ventricular tachycardia that is not hemodynamically significant. The patient associated with this finding is CRIM positive and initiated treatment early (≤ 1 month of age). This patient's conduction abnormality highlights the variable nature of the disease.

## 4 Discussion

Our study aimed to comprehensively evaluate cardiac outcomes in a large cohort of patients with IOPD enrolled in our Duke Pompe disease registry study. The analyses here have revealed differential cardiac outcomes based on timing of ERT initiation. The time to normalization of LVMI was faster in the early‐treated cohort. This underscores not only the effectiveness but also the rapidity with which early treatment normalizes cardiac hypertrophy in IOPD. To account for the potential of a lower disease burden at baseline (as is often the case with early initiation of treatment), we included baseline LVMI (first recorded LVMI) in our statistical model.

LVMI is only a single measure of cardiac hypertrophy, and it is important to wholistically evaluate additional parameters indicative of hypertrophy and cardiac function. Recent work at other institutions has demonstrated that cardiac function by way of echocardiograms (LVMI) and myocardial deformation (LV strain) normalizes after 1 year of ERT and remains stable [11]. A subset of patients, however, respond more slowly and/or fail to achieve complete normalization of LVMI and additional cardiac functional measures, despite treatment in infancy (especially when treatment begins later than 1 month of age). In addition to age and stage of disease at the time of ERT initiation, HSAT can impact the cardiomyopathy and ejection fraction response to ERT [22]. It is therefore important to consider the possibility of HSAT in the case of a patient who is not appropriately responding to ERT or who is experiencing a resurgence of their cardiomyopathy.

Early treatment was significantly associated with multiple improved cardiac parameters: LVMI was significantly lower in early‐treated patients and early treatment was significantly associated with a more rapid achievement of LVMI normalization; chamber dimensions showed significant improvements with early treatment including interventricular septal diameter in diastole and systole, and left ventricular posterior wall thickness in diastole and systole. Left ventricular hypertrophy, which has been consistently observed in IOPD based on EKG findings [2] is due to glycogen accumulation in cardiac muscle and leads to thickened interventricular septum and thickening of the left ventricular posterior wall [23]. Our study here noted that better outcomes, based on earlier treatment initiation, were observed in IVS and LVPW parameters.

Our findings support the benefit of early ERT initiation in IOPD, particularly for cardiac outcomes. The normalization of LVMI is a critical benefit, with early treatment leading to more rapid normalization. The positive effects were consistent across multiple cardiac parameters and analysis approaches, providing robust evidence for early intervention.

Importantly, prior to the analyses presented in our study, there had been limited information on how earlier ERT initiation may alter the electrophysiological phenotype of patients with IOPD. This study is unique in its analysis of the timing of ERT initiation as the main factor in comprehensively analyzing echocardiogram and electrocardiogram results of treated patients with IOPD. The strengths of this study include: the inclusion of 22 CRIM negative patients in the overall cohort, a comprehensive statistical approach with appropriate models for different outcome types, careful handling of repeated measurements using mixed‐effects models, and appropriate stratification by CRIM status. Furthermore, regarding the LVMI time to normalization analysis, an enhanced Cox model was used and incorporated additional variables—particularly LVMI baseline.

The limitations of this study include: wide confidence intervals in some analyses, small sample size in the early treatment group (*n* = 10) with an even smaller subgroup of CRIM‐negative early treatment (*n* = 2), limiting statistical power; and limited observations for certain abnormalities, particularly in the early treatment group. This also led to small subgroups for the CRIM status stratified analyses—15–18 patients per outcome in the CRIM negative subgroup and 16–23 patients per outcome in the CRIM positive subgroup. Certain cardiac outcomes (2 continuous outcomes and 6 binary outcomes) required simpler statistical models due to convergence issues, leading to potentially less precision. Six additional binary cardiac outcomes had insufficient data to be analyzed. Furthermore, the receipt of ITI and development of HSAT are further clinical confounders that would have been worth exploring but were not possible based on the small subgroup sample sizes that would result. The *p*‐values for all these characteristics (ITI, CRIM status, HSAT) indicate no statistically significant differences between early and late treatment groups, and the small sample sizes would make additional subgroup analyses underpowered and prone to type II errors. Multiple statistical tests were performed without formal adjustment for multiple comparisons, which could increase the risk of Type I errors (false positives). Lastly, to our knowledge, 8 individuals in the cohort are deceased, which may lead to survivorship bias (a form of selection bias). Results should be interpreted with appropriate caution, especially for findings with borderline statistical significance. Another limitation of this study is that all analyzed echocardiogram and EKG data were acquired clinically and therefore include varying intervals and varying lengths of longitudinal follow‐up, but this was accounted for in the statistical methodology used. Another limitation is that our study did not factor in the cumulative dose of ERT. Lastly, in the time‐to‐event analysis of LVMI normalization, we have not necessarily captured each patient at the exact time of initial normalization as it may have occurred earlier than the time of their clinical echocardiogram. Furthermore, there were insufficient data to assess LVMI in all individuals at approximately 1 year post‐ERT initiation.

Our study demonstrated that overall, echocardiogram and electrocardiogram parameters appear to have a higher frequency of normal values for age when ERT is initiated earlier; however, the data collection was limited in its retrospective and clinical nature. A better understanding of the specific mechanisms of the conduction abnormalities likely occurring secondary to glycogen accumulation may guide future therapeutic strategies—either earlier ERT administered in utero (prenatally) or new therapeutic approaches such as gene therapy.

## Author Contributions

J.L.C., A.P.L., P.S.K. conceived of the study. E.R.‐R. and V.G.S. conducted data acquisition and entry. M.M.B., A.P.L., and J.L.C. conducted data analysis. P.B.S. provided biostatistical guidance for data analysis. J.L.C. wrote the initial draft of the manuscript, and all authors contributed to editing the manuscript. The authors would like to acknowledge Eric Monson, PhD for his assistance with the manuscript's data visualization and Tracy Truong, MS for her guidance on statistical aspects of the study.

## Funding

This work was funded in part by a grant from the Children's Health and Discovery Initiative at Duke University. This research was supported in part by a grant from Sanofi and in part by the Lysosomal Disease Network (LDN). LDN (2U54NS065768) is a part of the National Center for Advancing Translational Sciences (NCATS) Rare Diseases Clinical Research Network (RDCRN). RDCRN is an initiative of the Office of Rare Diseases Research (ORDR), NCATS, funded through a collaboration between the NCATS, the National Institute of Neurological Disorders and Stroke (NINDS), and the National Institute of Diabetes and Digestive and Kidney Diseases (NIDDK). Jennifer L. Cohen is funded by a career development award, K23HD113824 through the National Institute of Child Health and Human Development (NICHD) and is also funded by the Y.T. and Alice Chen Pediatric Genetics and Genomics Research Center.

## Ethics Statement

The individuals included in this study are consented through Duke University's IRB‐approved study, Determination of Cross‐Reactive Immunological Material (CRIM) status and Longitudinal Follow‐up of Individuals with Pompe Disease (Pro00001562).

## Consent

The individuals included in this study are consented through Duke University's IRB‐approved study, Determination of Cross‐Reactive Immunological Material (CRIM) status and Longitudinal Follow‐up of Individuals with Pompe Disease (Pro00001562).

## Conflicts of Interest

Jennifer L. Cohen was a consultant for Bayer Healthcare Pharmaceuticals and served as an advisor for an Advisory Board for Sanofi/Genzyme. P. Brian Smith is a consultant for Syneos Health, Biocryst, and Tellus. Priya S. Kishnani has received research/grant support from Sanofi Genzyme and Amicus Therapeutics; received consulting fees and honoraria from Sanofi Genzyme, Amicus Therapeutics, and Asklepios Biopharmaceutical Inc. (AskBio); is a member of the Pompe and Gaucher Disease Registry Advisory Board for Sanofi Genzyme, Pompe Disease Advisory Board for Amicus Therapeutics, and Advisory Board for Baebies; has held equity in Asklepios Biopharmaceuticals and may receive milestone payments related to that equity in the future.

## Supporting information


**Table S1:** Genotypes of individuals included in the study cohort.

## Data Availability

The data that support the findings of this study are available on request from the corresponding author. The data are not publicly available due to privacy or ethical restrictions.
